# Supplementary material for: Temperature time series analysis at Yucatan using natural and horizontal visibility algorithms
Source: PLoS One. 2019 Dec 19;14(12):e0226598. doi: 10.1371/journal.pone.0226598 (PMC6922406; doi:10.1371/journal.pone.0226598)
Supplement: Attachment — Submitted filename: Response to Reviewers.pdf [file pone.0226598.s004.pdf]

# Dear Reviewers and Editor

We appreciate your comments and suggestions. They helped us a lot to improve the article and also to be able to give a clearer understanding of the idea that we want to communicate to the readers. We hope that with these corrections the points we want to release will become clearer. We thank you for your time and patience.

About the main observation that you mentioned about the writing, we decided to send the document to  a writing style correction agency to ensure the quality of the manuscript. We attach the certificate that was given to us along with the corrections.

The letter is organized as follows: First  the response to the editor and then the response to the reviewers. Each answer is a letter for each one. We include the editor's comment (in bold) and the answer begins with an $A$.

Once again we thank you for your support. We look forward to your comments

## Reviewer 2

**Thank you very much for the detailed answers to all issues that is raised by the reviewers. I have a small question that bothers me. Since you use statistical testing procedures, are there any assumptions like normally distributed variables? Please correct some typos that you add in this revision. Especially p-value$<0.05$ parts.**

**A.** We thank you very much for your comments, in fact to apply this test the data has to be independent and normal. The independence of the data is obtained since the measurements were made in each city with its own measuring instrument and there is no relationship from one city to another in the measurement process.

For the normality of the distribution of the data a statistical test was made. Usually, Kolmogorov-Smirnof test is used, but since there are only 21 data, Shaphiro-Wilk is used, which applies to samples of 50 records or less. We obtain the following values of p. The green color indicates that $p > 0.05$ wich indicates normally distributed data. If $p < 0.05$ we have non-normal data (Yellow color).

| | **Measurement** | **Merida** | **Progreso** | **Sisal** | **Telchac** | **SierraP** |
|---|---|---|---|---|---|---|
| **General** | Media | 0.00330591 | 0.00054404 | 0.0044705 | 0.00869545 | 0.00244482 |
| | DevStd | 0.12769559 | 0.03318864 | 0.22441483 | 0.02037118 | 0.98413329 |
| | ApEnt | 0.73681198 | 0.57449912 | 0.64037782 | 0.37098163 | 0.22432368 |
| | DFA | 0.25759864 | 0.77971655 | 0.66232862 | 0.62602547 | 0.48277831 |
| **NVA** | mean deg | 0.31455408 | 0.34683294 | 0.15390243 | 0.3379569 | 0.54944949 |
| | geodescics | 0.12160723 | 0.16405199 | 0.15892265 | 0.15281428 | 6.50E-05 |
| | clustering | 0.58601159 | 0.09595623 | 0.22182491 | 0.00849342 | 0.85359038 |
| | assortativity | 0.08866683 | 0.69267346 | 0.23608482 | 0.7582951 | 0.74569767 |
| | Laplacian Ener | 0.08572239 | 0.79764814 | 0.39048238 | 0.00037081 | 0.05938504 |
| | Shannon Ent | 0.24241821 | 0.07347954 | 0.06860002 | 0.22738198 | 0.55462299 |
| **HVA** | mean deg | 0.53432317 | 0.25306173 | 0.20985021 | 1.46E-05 | 0.37940119 |
| | geodescics | 0.07481793 | 0.02790849 | 0.24040644 | 0.19073564 | 0.6971402 |
| | clustering | 0.40871383 | 0.43748458 | 0.03630906 | 1.18E-07 | 0.66769125 |
| | assortativity | 0.99948168 | 0.33598857 | 0.53141366 | 0.00404848 | 0.11568569 |
| | Laplacian Ener | 0.87650306 | 0.2982707 | 0.30343033 | 1.80E-09 | 0.18310141 |
| | Shannon Ent | 0.65826936 | 0.13908803 | 0.39783225 | 2.38E-06 | 0.05017746 |

Given this analysis, we have decided to include only cities and measurements that do meet normal data (green in rows and columns). So we will only stay with:

Cities: Merida, Progreso, Sierra Papacal and Sisal

Measurements: ApEnt and DFA, Mean Degree, Assortativity, Laplacian Energy, Shannon Entropy.

This ensures us to continue comparing all measurements with all forms of transformation algorithm and among all cities since they all belong to normal distributions.

We included mean and standard deviation of the temperature in Table 1 but only as a reference to the readers, but these were not included in the t-test analysis.

Also, without the variables and measurements we took away, the paper is much more clear. Thank you for this important observation.

## Reviewer 3

Thanks for the feedback. As we mentioned at the beginning, all the errors and misunderstandings in writing were corrected, as well as the typos and symbols that were not correctly reflected in the PDF. Next we add the concrete answers to the corrections that are not related to typos, writing or contradictions between what is shown in the tables and what is commented in the manuscript:

**Figures 5 and 6: I still have a hard time understanding the circular plots. Apart from the visual interpretation (or lack on it), I don't understand why the authors are trying to infer general characteristics of the time series from a sample of only five days (where the authors even admit that the five days were chosen to illustrate a specific point). I would just omit these two plots to avoid confusing the reader.**

**A.** The entire “Comparing Extreme cities” subsection was omitted because we realized that it only confuses the reader instead of generating more clarity or information that supports the main conclusions. The images were also removed leaving only the time series to show the part we need to be clear in the readers.
