# Supplementary material for: Temperature time series analysis at Yucatan using natural and horizontal visibility algorithms
Source: PLoS One. 2019 Dec 19;14(12):e0226598. doi: 10.1371/journal.pone.0226598 (PMC6922406; doi:10.1371/journal.pone.0226598)
Supplement: Attachment — Submitted filename: referee report_PONE-D-19-14940.docx [file pone.0226598.s002.docx]

REPORT OF A REFEREE ON PAPER

**Manuscript Number:** PONE-D-19-14940

**Title**: Temperature Time series analysis at Yucatan using Natural and Horizontal Visibility Algorithms

Dear editor,

The paper is aimed to use network analysis tools to extract more information from monthly temperature series. Methods that are used in this study are already existed methods but not all applied to temperature series. The authors use a different approach to analyze temperature series. I think it is a new tool in climate studies, but authors need to explain the findings more, and focus on what the network analysis offers that other statistical tools such as clustering cannot provide.

There are some parts that can be explained better to increase the quality of paper.

1. What kind of gain that can be observed by using HVA and NVA compared the any classification or clustering should be given clearly.
2. Why are data collected in every two minutes, and then averaged in 10 minutes period, and then analysis done on a monthly basis? It is not clear.
3. On page 6, first sentence mentions the bell max at k=40 and 33. How these values can be interpreted? What is the meaning of having k=40 or 33? Please give some feedback.
4. Why on the first 5 days of My 5, 2015 chosen to use the network?
5. On page 7, line 173, it is written that “NVA has more connections then HVA”. What is the advantage/disadvantage of having more connections?
6. On Figure 5 there is a star shape with 6 points for Merida. How one can interprets these 6 points? Also, there are few connections on the middle. What is the meaning of this?
7. On page 8 line 203 “my” should be “may”.
